# iRefScape. A Cytoscape plug-in for visualization and data mining of protein interaction data from iRefIndex

**DOI:** 10.1186/1471-2105-12-388

**Published:** 2011-10-05

**Authors:** Sabry Razick, Antonio Mora, Katerina Michalickova, Paul Boddie, Ian M Donaldson

**Affiliations:** 1 The Biotechnology Centre of Oslo, University of Oslo, P.O. Box 1125 Blindern, 0317 Oslo, Norway; 2 Biomedical Research Group, Department of Informatics, University of Oslo, P.O. Box 1080 Blindern, 0316 Oslo, Norway; 3 Department for Molecular Biosciences, University of Oslo, P.O. Box 1041 Blindern, 0316 Oslo, Norway; 4 Scientific Computing Group, University of Oslo, P.O. Box 1059 Blindern, Oslo, Norway

## Abstract

**Background:**

The iRefIndex consolidates protein interaction data from ten databases in a rigorous manner using sequence-based hash keys. Working with consolidated interaction data comes with distinct challenges: data are redundant, overlapping, highly interconnected and may be collected and represented using different curation practices. These phenomena were quantified in our previous studies.

**Results:**

The iRefScape plug-in for the Cytoscape graphical viewer addresses these challenges. We show how these factors impact on data-mining tasks and how our solutions resolve them in a simple and efficient manner. A uniform accession space is used to limit redundancy and support search expansion and searching on multiple accession types. Multiple node and edge features support data filtering and mining. Node colours and features supply information about search result provenance. Overlapping evidence is presented using a multi-graph and a bi-partite representation is used to distinguish binary and n-ary source data. Searching for interactions between sets of proteins is supported and specifically includes searches on disease-related genes found in OMIM. Finally, a synchronized adjacency-matrix view facilitates visualization of relationships between sets of user defined groups.

**Conclusions:**

The iRefScape plug-in will be of interest to advanced users of interaction data. The plug-in provides access to a consolidated data set in a uniform accession space while remaining faithful to the underlying source data. Tools are provided to facilitate a range of tasks from a simple search to knowledge discovery. The plug-in uses a number of strategies that will be of interest to other plug-in developers.

## Background

The interaction reference index (iRefIndex) consolidates protein interaction data from ten databases including BIND [1], BioGRID [2],CORUM [3], DIP [4], IntAct [5], HPRD [6], MINT [7], MPact [8], MPPI [9] and OPHID [10]. The iRefIndex [11] uses a unique consolidation process: each interactor's amino acid sequence and taxonomy identifier are used to assign a universally accessible hash-key to each distinct interactor and interaction. This facilitates identification of redundant interaction records (and interactors) at search time regardless of the protein accession system used to construct a query. The resulting index is available as a tab-delimited file in MITAB format (http://irefindex.uio.no) and more recently, we have described a web-interface [12] to this resource that allows retrieval of data using a rich set of interaction record features. In this paper, we describe the iRefScape plug-in for Cytoscape [13] that facilitates retrieval, visualization and navigation of this data set in a graphical environment [14].

**Figure 1:**
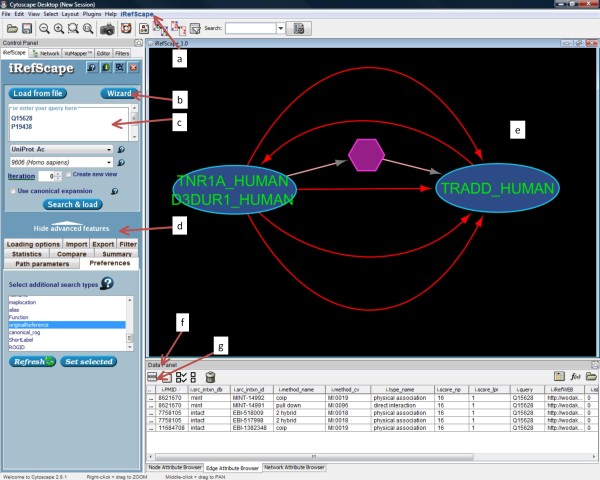
**Basic layout of the plug-in and distinctive features**. Plug-in functions are available in the iRefIndex menu (a), the wizard (b), the search area (c), the advanced features area (d), and by right-clicking on any node or edge in the graphical view area (e). Tabs in the Data panel (f) show Node Attributes and Edge Attributes. The user may choose which attributes appear in these panels using the Attribute selector icon (g). Two protein nodes appear as ovals. Each has a distinct amino acid sequence. A multi-graph representation allows multiple edges between these two nodes where each represents source data supporting some relationship between the two. A bipartite representation is used to distinguish n-ary interaction records (hexagons with pink edges) and their members (adjacent nodes) from binary interaction records. The edges selected using the mouse are shown in red and their properties will be visible from the attribute browser.

The universal identifier system for proteins and interactions is central to understanding the plug-in. Each protein interactor is assigned a "Redundant Object Group Identifier" or ROGID using its primary amino acid sequence, NCBI taxonomy identifier and the SHA-1 digest algorithm [11,15]. ROGIDs for interactors are, in turn, used to construct an SHA-1 hash key for the interaction record itself - a "Redundant Interaction Group Identifier" or RIGID. These keys are global (they can be generated by anyone) and they serve to group related interaction information. Figure 1 shows two interactor nodes joined by multiple edges in the iRefScape view; each edge represents a distinct interaction record but all these edges share the same RIGID - each record represents some experimental result that supports the idea of some kind of relationship between the same two proteins.

Using interaction data can be difficult due to a number of challenges that are distinct to this data type: multiple accession systems for protein data, high redundancy between interaction databases, multiple methods for complex representation, the scale-free nature of interaction networks and the resulting impact on visualization. Some of these issues have been discussed at length in our previous papers [12,16,17]. Here, we focus on the solutions employed by the iRefScape plug-in to help the user deal with these challenges. These solutions are grouped under four major headings: data content and delivery, data retrieval, data presentation and data mining and navigation. Critical and distinctive features of the plug-in are listed in Table 1.

**Table 1:** Outline of solutions employed by the iRefScape plug-in

Challenge/Need	Solution	**See**:
**Data content and delivery**		

Multiple source databases with redundant and non-homogenous data.	Consolidation and normalization of data.	1.1

Need to retrieve a large dataset for subsequent visualization and filtering. Need for a private and static data set.	Operate on a local data set. Use of file system and collision-proof identifiers for storage and retrieval of meta-data.	1.2

**Search**		

Need to support searching on multiple accession systems for proteins.	Use of universal ROGIDs and RIGIDs.	2.1

Differential choice of splice isoforms when curating interaction data.	Use of canonical expansion during search.	2.2

First order neighbours do not accurately reflect the neighbourhood of a protein.	Use of neighbourhood completion for query results.	2.3

**Visualization**		

Need to track search result provenance.	Use of features and colour to indicate result level. Use of "Grid" layout for initial results.	3.1

Need to visualize all evidence supporting a given interaction.	Use of a multigraph representation	3.2

Need to distinguish between binary and n-ary interaction data.	Use of bipartite model to visualize n-ary data.	3.3

Need to view/filter search results on multiple node and edge attributes.	Distribution of rich and normalized attribute set. Advanced filter function. Dedicated "between-nodes" search.	3.4

**Data discovery and navigation**		

Need to identify potential spoke-represented complexes.	Dedicated search tool.	4.1

Need to search on groups of genes related to a disease.	Dedicated disease-group search.	4.2

Need to explore connection between two groups of genes.	Use of synchronized adjacency-matrix viewer.	4.3

Need for interoperability with other plug-ins.	Export functionality.	4.4

Need for user to navigate a complex interface.	Use of wiki-documentation, in-line help and macros.	4.5

## Results

### 1. Data content and delivery

#### 1.1 Consolidation and normalization of data

Version 8.0 of the iRefIndex accompanies the release of iRefScape (1.16) described in this paper (see Methods). The final data set is based on 1,057,642 source interaction records but is only composed of 480,368 distinct interactions (RIGID's) involving 86,757 distinct protein interactors (ROGIDs). Version 8 includes 2,580 updates for protein database accessions, 4,748 corrections to malformed accessions, 19,278 corrections to taxonomy identifiers and more than 8,084 resolutions of ambiguous identifiers. Terms describing interaction type, interaction detection method and interactor detection method were normalized to PSI-MI ontology terms. Full statistics for each release are provided at http://irefindex.uio.no. Corrections made to source data were essential for legacy databases (such as BIND, MPact and OPHID) that are no longer updated.

The highly redundant nature of protein interaction data from multiple sources has been described previously [12]. Central to iRefScape is the use of universal identifiers to group together related interaction records. In the iRefScape view, each node represents a distinct interactor having a unique sequence and taxon identifier. Multiple edges may exist between the same two nodes where each edge represents an individual source record from some database and provides some experimental observation (or prediction) that supports some kind of relationship between the two adjacent nodes. Each of these edges (between the same two nodes) will share the same RIGID (redundant interaction group identifier). Figure 1 shows an example.

#### 1.2 The iRefScape plug-in operates on a local dataset

Installation of the iRefScape plug-in is a two step process. First, the plug-in itself is installed using the Cytoscape "plug-in manager". Second, the plug-in queries the user for a permissible location to store data and then retrieves the entire iRefIndex dataset via File Transfer Protocol (FTP). An "Installation and index handler" module executes these functions with a minimum of user intervention.

All iRefScape operations are performed on this local dataset. We chose this local approach (as opposed to web-services) for a number of reasons. Data retrieval is independent of connectivity over a network and it is not subject to communication overhead or server outages; therefore, it is fast and reliable. Local search is also private; a concern among commercial users. A local dataset, being static, ensures that results can be reliably reproduced and compared over the course of a study where multiple search strategies may be examined. Finally, a local dataset also affords the possibility for the plug-in's functions to operate over an entire interactome and a large set of attributes: something that would be impractical if the data were not already available locally. In addition, the user can include and search on a private set of attributes for any node or edge in the iRefIndex data set.

We found that this approach was a feasible option for data delivery in terms of both required disk space and search times. Download and indexing of the 362 MB package for release 8.0 of iRefIndex required less than 10 minutes using a remote, 10 Mbps internet connection. The data package is compressed on arrival and is dynamically uncompressed as required by user searches (see Data access optimizations in Methods). Thus, a search will take less time to return results after it has been performed once. For example, it takes 9 seconds to retrieve interactions involving UniProt:Q39009 and UniProt:Q9ZNV8 for the first time while the same operation subsequently takes only 2 seconds.

### 2. Search

#### 2.1 iRefScape search supports multiple accession types

**Figure 2:**
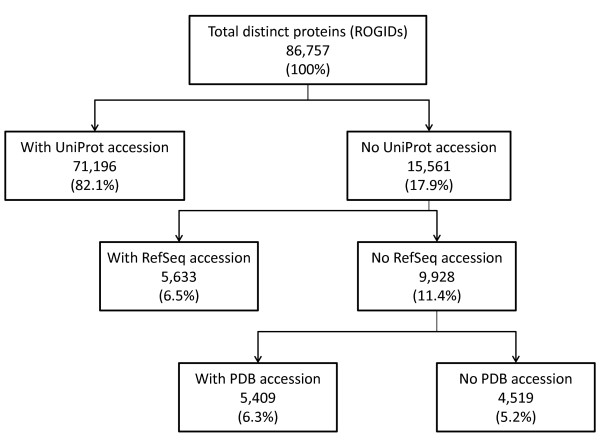
**Protein identifier types and reachability**. This figure provides the availability of accession types for proteins found in iRefIndex version 8.0. There were 86,757 ROGIDs which refer to unique primary amino sequence and NCBI taxonomy identifier combinations. Out of these, only 82.1% can be directly mapped to a UniProt/KB accession. Thus there were 15, 561 ROGIDs that could not be mapped to a UniProt/KB. Out of the 15,561 that could not be mapped to a UniProt/KB, 9,928 could not be mapped to a RefSeq accession either. Of the 11.5% of proteins in iRefIndex that could not be mapped to a UniProt or a RefSeq accession 5,409 had PDB identifiers. Finally 4,519 ROGIDs (5.2% of all ROGIDs) were not reachable using queries with any of these accession types but could be found using ROGIDs or other accession type searches.

An identifier for a protein (or a list of identifiers for multiple proteins) may be entered via a query box or from a file. Multiple accession-types are supported including those from RefSeq and UniProt. Entrez Gene IDs and official gene symbols are converted to accessions for their corresponding protein products. This functionality is driven by lookup tables where the user's query retrieves a pre-computed redundant object group identifier (ROGID) [11]. Since all interactors from all interaction records are mapped to ROGIDs, the user's query is guaranteed to return all protein interactors with identical sequences regardless of the accession system used to describe the interactor in the original interaction record. Example searches are provided with the iRefScape documentation. A user's choice of accession type (e.g. UniProt versus RefSeq accessions) can have an impact on their search results because there is no single protein identifier type that is available for all protein interactors in iRefIndex. The reachability graph in Figure 2 shows that 82% of all distinct interactors have a UniProt accession. Twelve percent do not have a UniProt accession but do have a RefSeq or PDB chain accession. All other interactors (5.2%) can be retrieved using a catchall "Xref" search type or by searching directly for ROGIDs generated by the user from a protein's amino acid sequence. A wizard utility allows the user to generate a ROGID for an entered amino acid sequence. Protein accessions found in source databases are updated to their latest versions if they have been retired. However, many of these retired accessions can still be searched for using the *originalReference *search type.

#### 2.2 Canonicalization and searching

**Figure 3:**
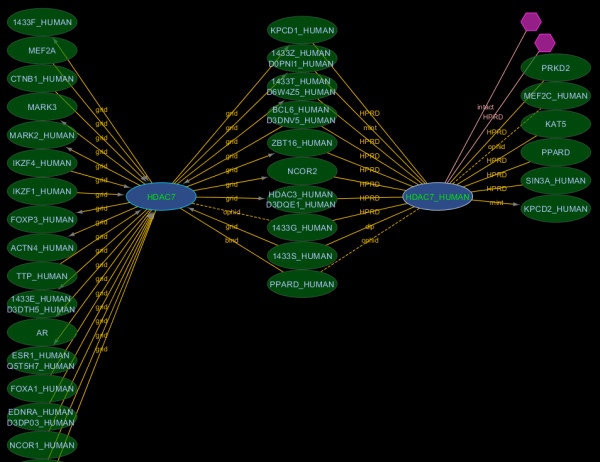
**Canonical expansion**. A search for first order interactors of Entrez GeneID 51564 (HDAC7) with canonical expansion of the search term returns interactors for two isoforms of HDAC7 (blue nodes). Searching without canonical expansion would only return interactors for the node labelled HDAC7 (corresponding to RefSeq: NP_056216) because the sequence for HDAC7_HUMAN (UniProt: Q8WUI4) was not among the splice-isoforms in the Entrez Gene record at the time of iRefIndex build 8.0. However, the Q8WUI4-5 splice isoform was identical to NP_056216 so both interactors were mapped to the same canonical group. Manual inspection of the source records found no evidence for the involvement of any particular splice isoform of HDAC7 so the interaction data for both forms shown can be merged safely.

The canonicalization process groups together multiple proteins that are all products of the same or related genes and then chooses one of these proteins to be the canonical representative for the whole group (see Methods). Figure 3 illustrates how search results differ if the user chooses to expand their query to include all sequences belonging to the canonical groups in their initial query. Nodes belonging to the same canonical group can be identified and manually grouped together using the i.canonical_rogid node feature. Different nodes belonging to the same canonical group may indicate interaction data that is specific to different splice isoforms or simply that the source publication was ambiguous with respect to the splice isoform involved [16]. Sixty-four percent of all interactors can be retrieved using an Entrez Gene ID (80% when canonical search expansion is used).

#### 2.3 Neighbourhood completion

**Figure 4:**
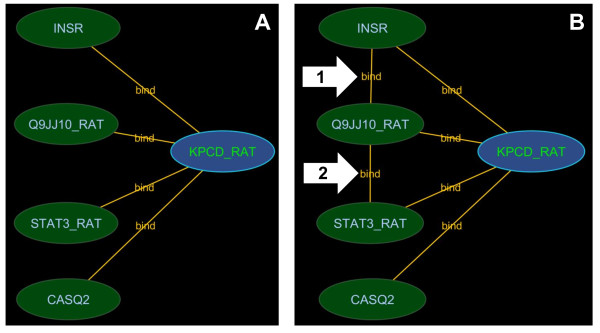
**Neighbourhood completion**. This figure shows search results for P09215 without neighbourhood completion (A) and with neighbourhood completion (B). The neighbourhood completion search shows not only direct interactions involving the query but also how the interacting proteins interact with each other. Type A results are commonly returned by interaction search interfaces. The arrows with number 1 and 2 show the edges retrieved when neighbourhood completion was performed.

A query for a protein will return its nearest neighbours. However, interactions between these first order neighbours will not be returned by default unless the user opts to complete the neighbourhood once the first order neighbours have been returned. Figure 4 illustrates the effect of using this feature. The underlying search is effectively a search for all interactions that are known to occur between interactors in the current view. This expanded-search operation can be expensive and further justifies the need to operate on a local data set.

### 3. Visualization

#### 3.1 Search result provenance - differentiating between query and neighbour nodes

A common difficulty working with graphical data is distinguishing between nodes matching search parameters from their interactors. We have addressed this issue using a number of visual cues and node/edge features.

Nodes that match search parameters are coloured blue while direct interactors are green (Figure 3). Colours are mapped by the Cytoscape VizMapper using the i.order node feature and can be customized by users. As new nodes are added to the view by subsequent queries, they are assigned incremental i.order values and new colours corresponding to new results from that search set. In addition, the search terms responsible for returning a node in the current view are listed under the node feature i.query (Figure 3).

The "Grid" layout was selected as the default arrangement for all new search results. This algorithm was the fastest amongst those investigated for reasonably sized networks (see Layout and visual properties in Methods). Users are likely to alter the layout while viewing search results so these alterations are preserved as subsequent search results are drawn; only newly introduced nodes are arranged in a distinctive grid layout making them easier to locate in a graphical view.

#### 3.2 A multigraph view shows multiple supporting pieces of evidence

Search results are presented as a multi-graph (i.e., multiple edges are allowed between two nodes). Each node represents a distinct protein sequence from some organism (ROGID). Each edge joining the same two nodes will have the same RIGID (Redundant Interaction Group Identifier) because each represents an experimental observation supporting the same *interaction*. However, the experimental type and interaction type for each edge in this group may be different. Each edge is listed in the edge attribute browser along with source database and supporting publication details allowing easy linking to the original data. The edge attribute browser may also be used to sort and select (or remove) edges with specific attributes. For example, the only source of predicted interaction data in iRefScape is OPHID - these edges may be located and high-lighted using the edge attribute for source database (i.src_intxn_db). As a special precaution, OPHID edges are drawn using dashed lines so they are visually distinguishable from other data. All other data in iRefScape is based on experimental observation. Again, edge attributes corresponding to experimental method (i.method_name and i.method_cv) may be used to sort, select and high-light specific edges.

This multi-graph representation can become visually cluttered. We therefore included the ability to collapse multiple edges, leaving only a single (randomly selected) edge in the view panel (see iRefScape menu/View Tools/Toggle selected multi-edges). The edge-toggle operates using the edge attribute "i.flag" so the user can manually alter the edges that are retained in the toggled view. Collapsed edges can be re-expanded by selecting the same edge-toggle function again.

Multiple edges between nodes represent a mixture of records that are either duplications within or between databases or that are multiple evidences for relations between the same two proteins. These two cases are easily distinguished in iRefScape by examination of edge attributes (for example, i.PMID" and i.method_name). The observed two-fold redundancy in interaction data underscores the need for visual tools that allow the user to visually manage related data.

#### 3.3 A bi-partite graph representation highlights n-ary data

The term "n-ary data" (and sometimes "complex data") is used to refer to interaction records that contain more than two protein participants. This term distinguishes such records from binary interaction records where only two interactors are listed. Multi-protein interaction records are used by some databases to capture the results of certain types of experiments where a physical association is detected between a number of proteins but where the presence or absence of a direct physical interaction between any given pair of proteins in the set cannot be determined (for example, results from an experiment where some tagged "bait" protein is used to co-immunoprecipitate a collection of proteins from a cellular extract). As such, n-ary data is fundamentally different from binary data and we have preserved this difference in iRefScape. N-ary records are represented by hexagonal *pseudonodes*. Protein nodes (ovals) adjacent to this node represent members of the record (example in Figure 1). Pseudonodes in a view can be selected and sorted using the node attribute called i.pseudonode.

Of the 433,617 distinct interactions (RIGID's) in iRefIndex, only 15,056 (3%) have greater than two participants. However, these records involve 20,228 distinct proteins (ROGIDs) or 24% of the dataset. The bi-partite representation described here clearly distinguishes involvement of these proteins in "n-ary" interactions from involvement in binary interactions that include evidence for direct physical interactions.

Users should be cautious in reading meaning into the labels "binary" and "n-ary" data. N-ary data does not necessarily constitute evidence for a biological complex: only that a group of proteins were observed together in some experimental result. Likewise, binary data does not necessarily constitute evidence for a direct physical interaction and in some rare cases may even be a spoke representation of a complex (see section 4.1). In all cases, it is important to examine the experimental type and/or the original publication before drawing such conclusions. Links to abstracts are provided in the interface as an edge attribute (i.PMID).

#### 3.4 Node and edge features

A large number of node and edge attributes are included with search results to facilitate further filtering and visualization of data. A complete list of these features can be found on the iRefScape wiki site [14]. Node attributes include accessions from different databases, taxonomy identifier, and overall degree in the consolidated iRefIndex data set as well as in the present view (i.overall_degree and i.alive_degree respectively). Edge attributes include interaction type, interaction detection method, publication identifier, source interaction database and accession, and directionality (for bait-prey systems). Controlled-vocabulary terms and identifiers are provided where possible and many of the attributes allow linking out to external databases. Bibliometric scores (i.score_np and i.score_lpr) help the user to identify edges supported by multiple citations and distinguish edges supported only by high-throughput studies respectively.

Users can create filters for search results using Cytoscape's in-built edge and attribute filters. In addition, a complementary, advanced filter utility is included that allows the user to build up and visualize more complicated queries (see Advanced Features/Filter). Instructions are provided in the plug-in.

Nodes matching filter criteria reset an attribute called i.alive to TRUE. Another node attribute (i.alive_degree) is updated to reflect the degree of a node with respect to neighbours with i.alive set to TRUE. This feature is useful for identifying nodes in the view that are highly connected to a user-defined set of nodes. As a short-cut, the user may simply select a set of nodes and then select "iRefScape menu/View Tools/Select between nodes" to do the same thing. Nodes that are highly connected to the original selection can be selected and sorted on the i.alive_degree node feature.

### 4. Data discovery and navigation

#### 4.1 Identification of spoke-represented complexes

**Figure 5:**
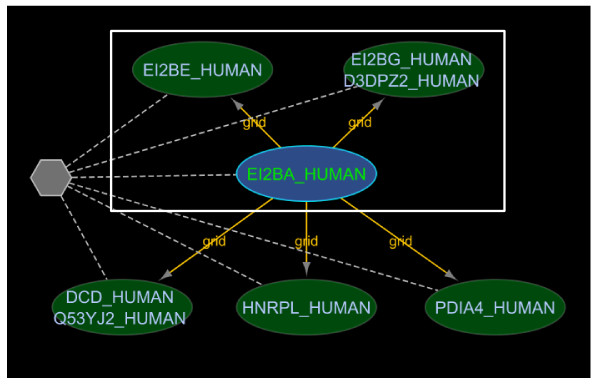
**Spoke represented complex**. This figure shows a possible n-ary interaction (six proteins in this case) represented using a spoke model where a series of binary interactions between one member chosen to represent the hub and each of the other members are created (see section 4.1 for more details). The "Show spoke-represented complexes" function in the iRefScape plug-in was used to identify such disassembled n-ary interactions and visually show the connection using a pseudonode (the gray hexagon in the figure) and a set of dashed edges. In the figure, the original five binary interactions from source databases are shown as continuous yellow edges between the six proteins. The data is from an affinity chromatography experiment [27] and is represented in both BioGRID and IntAct using a spoke-model. The white box indicates three subunits that overlap with the five genes encoding subunits of the translation initiation factor EIF2B. Mutations in any one of these subunits are associated with Leukoencephalopathy (OMIM #603896). The overlap between the Leukoencephalopathy gene list and the list of genes in this regenerated n-ary record are significant; however, the two lists would not normally be compared because the n-ary record appears as a series of binary records in interaction databases.

N-ary data is sometimes represented using a spoke model where a series of binary interactions between one member chosen to represent the hub and each of the other members are created [16]. This is commonly used in cases where the experimental method used to support the complex was a bait-prey, immunoprecipitation system: the bait is conventionally chosen as the central "hub" node. This spoke-representation makes it difficult to identify data that supports the idea of a protein complex (since it appears as a set of binary edges in the graph). iRefScape allows these cases to be identified in the current view using the "Show spoke-represented complexes" function in the iRefScape/View Tools menu. An example is shown in Figure 5.

iRefIndex 8.0 contains more than 77,048 distinct binary interactions (RIGID's) that may belong to spoke-represented complexes: these are sets of two or more binary interactions originating from the same database that all share the same publication reference, the same experimental method (known to produce n-ary data) and a common interactor (hub node). These binary interactions can be used to identify 21,251 cases in version 8.0 of iRefIndex where a source database may be using a spoke representation of a complex. The "Show spoke-represented complexes" tool draws a grey hexagon with dashed lines to each member of the potential complex. The user must still review the source publication to confirm that they contain evidence for a protein complex (as opposed to a set of separate binary interactions all demonstrated using the same bait protein).

#### 4.2 Disease group browsing

Genes that are associated with phenotypically related diseases are quite often associated in an interaction network [18]. We have constructed our own grouping of diseases and their related genes found in the OMIM [19] by grouping together OMIM record titles using regular expressions [20].

**Figure 6:**
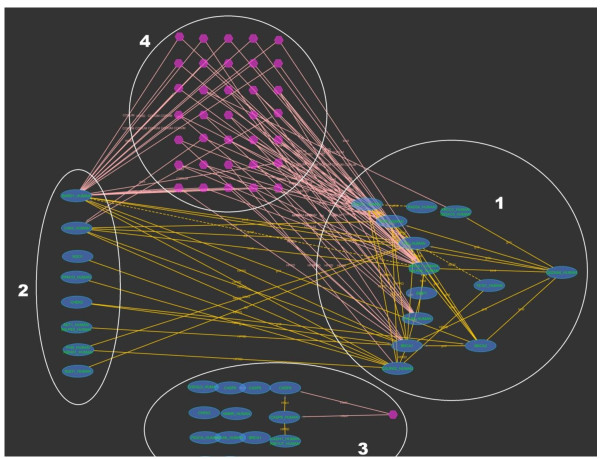
**Disease group browsing**. This example shows how iRefScape can be used to locate all the proteins connected with a certain disease and group them according to a selected property. The figure is not meant to show node or edge details, rather to demonstrate the search and the subsequent filtering. In this figure the results of the search type = digid for query "197" (breast cancer) with iteration set to 0 are shown. All the oval shaped nodes represent proteins from the genes in this group. The hexagons represent membership in the same n-ary interaction record. Subsequent to the search, a filter was applied to select all nodes which have the gene ontology function annotation "DNA binding". During the filtering, grouping was requested (by setting the "group mode on" under filters). This has resulted in three groups (1, 2 and 3 in the figure). Group 1 contains nodes with the annotation "DNA binding". Group 2 contains nodes which do not have the annotation "DNA binding" but have a direct interaction or a complex membership with nodes in Group 1. Group 3 contains the remaining nodes that do not belong to Groups 1 and 2. The set of nodes marked with number 4 shows the n-ary interaction records where at least one node with the annotation and one without the annotation were involved.

iRefScape supports searching for OMIM identifiers corresponding to gene-disease associations. Our disease group identifiers may also be searched in order to retrieve all proteins related to the same disease group as some OMIM identifier. Partial match searching of OMIM titles is also supported. Figure 6 shows the search results for digid 197 (Breast cancer).

Approximately 400 disease groups were constructed that contain two or more related human genes. These groups cover just fewer than 2000 genes, the majority of which have relevant interaction data in iRefIndex. A systematic survey of all statistically significant overlaps between disease groups and interaction data is being prepared

#### 4.3 Synchronized adjacency-matrix viewer

Due to the complexity of the protein interactome, it is difficult for the user to arrive at inferences by visual inspection of a graphical representation alone. We have implemented an adjacency matrix view to provide a very simple overview of the connectivity between two, user-selected groups of nodes. This adjacency matrix view is synchronized with Cytoscape's graphical view allowing users to move back and forth between the two representation types. In this view, members of group A and B appear as column and row headings respectively. If a node was found in both groups it will appear as a column and row heading. A cell at the intersection of a particular column and row contains information about the relationship between the two proteins appearing in the column and row headers. There are various possibilities:

A) When only direct interactions between a row protein and a column protein exist, a "¤" symbol appears in the intersection cell and the cell will be coloured red.

B) When only indirect interactions or co-complexes involving the row protein and column protein exist, the cell will be coloured blue. These are interactions involving an intermediate protein or both proteins found together in an n-ary record. The intermediate protein or the complex-node will be listed in the intersection cell.

C) When there are both direct and indirect interactions, the intersection cell will be coloured green. A "¤" symbol and all intermediate proteins or complex nodes will be listed in the intersecting cell.

D) When there are no direct or indirect interactions involving the row and column proteins, the cell colour is black. Entire columns or rows with only black cells will be hidden by default.

E) As a special case, when the row and column protein are the same (the protein is found in both groups), proteins directly interacting with this protein will be listed in the intersecting cell.

**Figure 7:**
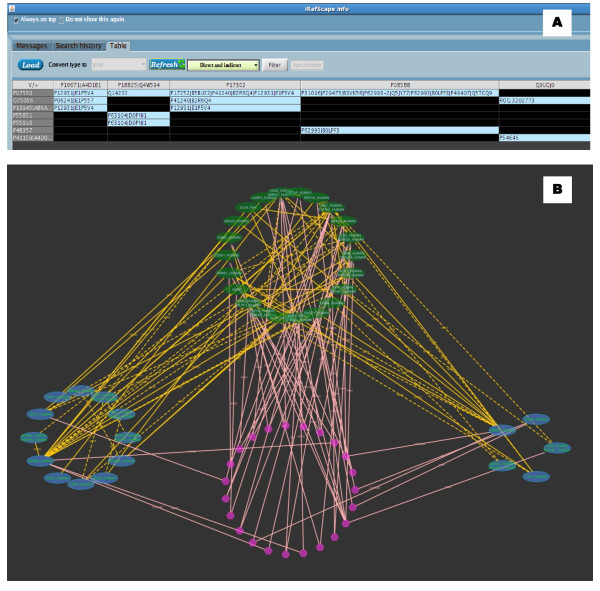
**Adjacency matrix view synchronized with graphical view**. A. Adjacency matrix view. Column headings are proteins related to heart disease and row headings are related to obesity [21]. B. Graphical view of this search in Cytoscape. The blue nodes represent the nodes from the two groups: heart disease (left) and obesity (right), Green nodes (top) represent the intermediary proteins between the two groups. Hexagonal nodes (bottom) represent n-ary data (possible complexes) having members from one or more of the three groups. As both views are synchronized, clicking on any cell in the tabulated view highlights the corresponding nodes in the graphical view.

Clicking on column/row headings or intersection cells will select and highlight nodes and/or relevant edges in the graphical view. Figure 7 shows an example comparing proteins related to heart disease (group A) and obesity (group B) taken from [21].

#### 4.4 Export and interoperability

iRefScape uses an internal integer identifier (iROGID) as the main node identifier (see the node feature ID in the Cytoscape interface). Other plug-ins may require that a specific identifier type (Entrez Gene ID or UniProt/KB accession) is used as the primary node identifier (e.g. BinGO requires Gene Ids [22]). As a solution to this interoperability problem, we have included an export feature which exports the iRefScape network using many popular identifier types. When performing the export, nodes which do not have the target identifier will be dropped. The export feature should be used with caution as the iROGID has a one-to-many relation with some identifier types (e.g. Entrez Gene Identifiers and canonical ROGIDs). For instance, when the iRefScape network is exported with Entrez Gene identifiers as the node identifier, several nodes represented by multiple iROGIDs (different splice isoforms) may map to a single Entrez Gene identifier. In these situations, several nodes will be collapsed to a single node and that node will have cumulative attributes from all of its constituent nodes.

#### 4.5 User Help

Help buttons (?) are distributed throughout the plug-in interface to provide context-relevant help. These buttons open the iRefScape info window. Help pages include macros ("Show me an example") that perform pre-recorded operations to help step users through the interface. Likewise, a "Wizard" button can be used to walk first-time users through simple searches. The iRefScape wiki provides additional details [14].

#### 4.6 Availability and updates

The Cytoscape Plug-in Manager (Plug-ins/Manage Plug-ins menu) can be used to install and update the plug-in. Installation instructions (including for manual download and installation) are available from the iRefScape wiki [14]. Users can check for updates to the plug-in data and help content using the iRefScape plug-in Wizard. Code is available upon request under a GNU-GPL license.

## Discussion

We have presented an interface that facilitates visualization, navigation and data mining on a consolidated protein interaction data set. Working with consolidated interaction data comes with distinct challenges that were identified in earlier studies. The features incorporated in the plug-in address many of these issues.

The iRefScape plug-in operates on a locally downloaded dataset organized around a uniform naming space for interactions and interactors. This approach handles redundancy in the underlying data set and supports searching on multiple accession types. Searches can be expanded to retrieve data relevant to all splice isoforms of a queried protein. Visual representation of the graph distinguishes between n-ary and binary interactions. A tool is provided to help identify spoke-represented complexes. Multiple sources of evidence for a protein-protein relationship can be individually visualized, examined and filtered on. Advanced filters are provided to identify nodes that are highly connected to a user-defined set of nodes. An adjacency-matrix viewer helps users pick out and explore links between two groups of user-defined nodes. Coupled together with direct-support for disease group searching, we imagine that these functions will be useful for exploring relationships within and between complex diseases.

## Conclusions

We have quantified different aspects of the data as justification for the features included in the plug-in and as a guide to expectations for the user as well as other developers of plug-in software. We hope this effort will benefit users not only as a search interface for consolidated interaction data but also as a tool for knowledge discovery.

## Methods

### 1. Development environment

The iRefScape plug-in was developed using guidance provided by the Cytoscape plug-in development tutorial. The development was carried out using the Java Development Kit 1.6 and NetBeans as an integrated development environment. Apache Commons was used for file download management.

### 2. Data consolidation and availability

Version 8.0 of the iRefIndex dataset was constructed as described in [11]. Data is made available under a Creative Commons license at the iRefIndex wiki site [23]. This site links to further information for each release describing source data, release statistics, feedback to source databases, and mapping of terms to the molecular interaction (MI) controlled vocabulary for interaction types and detection methods.

### 3. Canonicalization

Each protein interactor is assigned a canonical identifier (cROGID). This allows related proteins (say from the same or related genes) to be identified and possibly grouped together by the end user.

Entrez Gene records are associated with a list of zero or more distinct protein products (as indicated by the ROGIDs for these proteins). Entrez Gene identifiers were grouped together into related gene groups (RGGs) if they shared at least one identical protein product. Therefore each RGG has an initial list of distinct protein products encoded by at least one of its member genes and represented by a set of RefSeq protein records. This initial list was expanded to include (1) distinct proteins from UniProt proteins that were isoforms related to one of the proteins already existing in this list and/or (2) UniProt proteins that cross-referenced one of the Entrez Gene identifiers in the RGG. From this expanded list of proteins, one distinct protein was chosen to represent the canonical isoform for the entire list. If one of the proteins was a canonical sequence (as defined by UniProt [24]) then this was chosen as the canonical form. If two or more such proteins existed, the longest was chosen. If no canonical UniProt sequences existed, the longest protein sequence associated with the RGG was chosen.

### 4. Data access optimizations

We investigated three methods for data access including web-services and finally settled on a locally installed dataset for reasons given in the Results section [25]. The indices are imported as files with file names that contain steering information (how the file should be used). Frequently used indices are converted to Java serialized objects during plug-in installation and others are kept as text files. Additional information on how users may add custom-made indices is available on the iRefScape wiki site [14].

The first step of any protein identifier search is to find the corresponding ROGID for the protein using a pre-calculated identifier-to-ROGID index. ROGIDs are used to retrieve relevant interactions (RIGIDs). Finally, attributes for each node and edge are loaded into Cytoscape's attribute browser. Attribute data is stored locally at installation time in separate jar (Java archive) files for each node and edge. These files are named after the corresponding ROGID or RIGID (in its filename-safe version [11]). At search time, the relevant files are retrieved and decompressed and then used to construct the network view and associated attribute browser data. Since attribute files are compressed, the disk footprint on the client system is minimized. Since ROGIDs and RIGIDs are distinct, they can be used for collision-free and fast retrieval by the client's file system. Collision tests were carried out to confirm this - for release 57.14 of UniProtKB we found 8,750,947 distinct sequences and the same number of SHA-1 based keys. The CRC64 algorithm generated the same key for two different sequences only once (8,750,946 distinct keys).

### 5. Layout and visual properties

After evaluating 15 open-source layouts distributed with Cytoscape, we selected the "Grid" layout for initial presentation of search results as it was the fastest to draw (386 ms for a 424 node, 892 edge graph versus other layouts that ranged between 645 ms (degree-circle), 2 s (force-directed), 5 s (attributes-layout) and 351 s (jgraph-gem). We defined our own visual properties for nodes and edges using the Cytoscape VizMapper (iRef style). In some cases, this style refers to node and edge attributes imported at installation time; for instance, to help distinguish between protein nodes and pseudonodes representing n-ary data. Users can change this style in Cytoscape's VizMapper tab.

### 6. Synchronized adjacency table

The adjacency table view was constructed using a Java JTable [26]. Nodes are selected into two groups by the user (Group 1 and Group 2) with one group selected as column headings and the other as row headings. Each cell, being the intersection of a row and a column, is labelled with a "¤" symbol if the row and column proteins interact directly. If the row protein and the column protein interact through intermediate proteins, the intermediate proteins are included in the cell. The colour of the cell is decided according to the nature of the relationship, such as a direct interaction, relationships involving direct and intermediate interactions or interactions only involving intermediate nodes.

The query entered into the search box for Figure 7 was:

COMPARE{P08254,P08588,P16671,P17302,P18825,P78504,Q14524, Q9UGJ0,Q9Y4J8|O00253,O75056,P01189,P07550,P13945,P25874,

P29120,P32245,P37231,P41159,P41968,P48357,P52895,P55851,

P55916,P81133,Q15466,Q16620,Q86YN6,Q9UBU3}.

Iteration was set to 1, search type "UniProt_AC" and taxid = 9606(Human). During the loading process empty rows and columns were removed to make observation easier (selected by default). After the results were loaded the results were converted from ROGID to UniProt/KB to make the comparison with the original paper [21].

### 7. Testing

Testing of the iRefScape plug-in installation and operation was carried out using version 2.8.1 of Cytoscape running on several operating systems including Mac OS X, Windows Vista, Windows 7, and Red Hat Enterprise Linux. In addition to the test procedures carried out during the iRefIndex data release process, we have also carried out spot checks on iRefScape search results to ensure that they matched data from the primary source databases. The test cases and intended results were tabulated on the iRefScape wiki site [14].

## Authors' contributions

SR was responsible for all code development. AM developed functionality to find spoke-represented complexes. KM developed disease groups. PB and SR assembled iRefIndex data. IMD supervised the project. SR and IMD wrote the manuscript. All authors participated in testing and final preparation of the manuscript.
